# Supplementary material for: MetaRibo-Seq measures translation in microbiomes
Source: Nat Commun. 2020 Jun 29;11:3268. doi: 10.1038/s41467-020-17081-z (PMC7324362; doi:10.1038/s41467-020-17081-z)
Supplement: Supplementary file 10 — Supplementary Data 7 [file 41467_2020_17081_MOESM10_ESM.zip › File2/Confidence_High_Taxonomy/421124_out.krona.html]

Javascript must be enabled to view this page.

members
magnitude
magnitudeUnassigned
count
unassigned
taxon
rank

421124\_out

77

superkingdom
2
77

76
1239
phylum

186801
class
76

76
186802
order

76
family
541000

genus
1263
76

1
1262962
species

SRS074670\_contig\_number\_4796

1

SRS893369\_contig\_number\_8477
species
2293178

1
2293157
species

SRS104084\_contig\_number\_12906

165186

SRS011405\_contig\_number\_contig-100\_1684.105568SRS022524\_contig\_number\_9173SRS045195\_contig\_number\_248SRS048870\_contig\_number\_contig-100\_3.122690SRS054352\_contig\_number\_18507SRS142980\_contig\_number\_4748SRS144297\_contig\_number\_6360SRS893358\_contig\_number\_13716
species
8

1160721
species

SRS015579\_contig\_number\_14980SRS015694\_contig\_number\_18191SRS016018\_contig\_number\_6202SRS016629\_contig\_number\_3938SRS019030\_contig\_number\_16289SRS020394\_contig\_number\_15001SRS024009\_contig\_number\_11731SRS043701\_contig\_number\_contig-100\_16.74882SRS045826\_contig\_number\_21036SRS049712\_contig\_number\_18081SRS052027\_contig\_number\_16977SRS064276\_contig\_number\_33943SRS076804\_contig\_number\_20193SRS077641\_contig\_number\_15223SRS098073\_contig\_number\_12856SRS104636\_contig\_number\_28068SRS143722\_contig\_number\_contig-100\_372.33809SRS143876\_contig\_number\_20076SRS144714\_contig\_number\_14512SRS147139\_contig\_number\_36382SRS148424\_contig\_number\_34526SRS148874\_contig\_number\_15319SRS893170\_contig\_number\_9771SRS893279\_contig\_number\_15330SRS893373\_contig\_number\_2309SRS971276\_contig\_number\_34603
26

species

SRS019693\_contig\_number\_7857SRS893288\_contig\_number\_2941
1637508
2

25
species

SRS014855\_contig\_number\_9153SRS015854\_contig\_number\_contig-100\_1361.104381SRS016954\_contig\_number\_18224SRS017821\_contig\_number\_21717SRS018541\_contig\_number\_12645SRS018817\_contig\_number\_11970SRS019178\_contig\_number\_4379SRS020869\_contig\_number\_22916SRS024075\_contig\_number\_8663SRS024625\_contig\_number\_20594SRS042628\_contig\_number\_10808SRS045739\_contig\_number\_6890SRS046717\_contig\_number\_contig-100\_803.75720SRS063040\_contig\_number\_29216SRS063985\_contig\_number\_9557SRS075716\_contig\_number\_3893SRS098644\_contig\_number\_12190SRS142781\_contig\_number\_5248SRS143523\_contig\_number\_2047SRS148159\_contig\_number\_36026SRS148319\_contig\_number\_13213SRS893259\_contig\_number\_4936SRS893292\_contig\_number\_7343SRS893295\_contig\_number\_13145SRS893300\_contig\_number\_9600
41978

species

SRS012273\_contig\_number\_42136SRS1041133\_contig\_number\_6392
2293149
2

6
species

SRS014313\_contig\_number\_4447SRS051882\_contig\_number\_19533SRS077335\_contig\_number\_969SRS143780\_contig\_number\_13357SRS149181\_contig\_number\_30040SRS893270\_contig\_number\_22678
2293177

2293194

SRS097889\_contig\_number\_30586SRS101433\_contig\_number\_10283SRS142890\_contig\_number\_41430SRS149325\_contig\_number\_2728
species
4

1
976
phylum

1
200643
class

171549
order
1

family
815
1

816
genus
1

28116

SRS018313\_contig\_number\_1663
species
1
